# Explaining Cannabis Use by Adolescents: A Comparative Assessment of Fuzzy Set Qualitative Comparative Analysis and Ordered Logistic Regression

**DOI:** 10.3390/healthcare10040669

**Published:** 2022-04-02

**Authors:** Jorge de Andres-Sanchez, Angel Belzunegui-Eraso

**Affiliations:** Social and Business Research Laboratory, Campus Catalunya, University Rovira i Virgili, 43002 Tarragona, Spain; jorge.deandres@urv.cat

**Keywords:** adolescence, substance use, cannabis use, ordered logistic regression, fuzzy set theory, fuzzy set qualitative comparative analysis, Boolean functions

## Abstract

Background: This study assesses the relevance of several factors that the literature on the substance use of adolescents considers relevant. The factors embed individual variables, such as gender or age; factors linked with parental style; and variables that are associated with the teenager’s social environment. Methods: The study applies complementarily ordered logistic regression (OLR) and fuzzy set qualitative comparative analysis (fsQCA) in a sample of 1935 teenagers of Tarragona (Spain). Results: The OLR showed that being female (OR = 0.383; *p* < 0.0001), parental monitoring (OR = 0.587; *p* = 0.0201), and religiousness (OR = 0.476; *p* = 0.006) are significant inhibitors of cannabis consumption. On the other hand, parental tolerance to substance use (OR = 42.01; *p* < 0.0001) and having close peers that consume substances (OR = 5.60; *p* < 0.0001) act as enablers. The FsQCA allowed for fitting the linkages between the factors from a complementary perspective. (1) The coverage (*cov*) and consistency (*cons*) attained by the explanatory solutions of use (*cons* = 0.808; *cov* = 0.357) are clearly lower than those obtained by the recipes for nonuse (*cons* = 0.952; *cov* = 0.869). (2) The interaction of being male, having a tolerant family to substance use, and peer attitudes toward substances are continuously present in the profiles that are linked to a risk of cannabis smoking. (3) The most important recipe that explains resistance to cannabis is simply parental disagreement with substance consumption. Conclusions: On the one hand, the results of the OLR allow for determining the strength of an evaluated risk or protective factors according to the value of the OR. On the other hand, the fsQCA allows for the identification not only of profiles where there is a high risk of cannabis use, but also profiles where there is a low risk.

## 1. Introduction

Cannabis (hashish or marijuana) is probably one of the most commonly consumed drugs around the world [1,2]. Early adolescence is a critical period of neurodevelopment, with synaptic pruning and increased myelination occurring. These processes are essential for the optimal development of the cognitive, emotional, motivational, and sensorimotor functions [3]. The use of cannabis has significant health implications since its use impacts physically on the brain [2,4]. Thus, its use enables several psychological disorders, such as dependence, depression, or psychotic symptoms, which must be outlined [5,6]. Likewise, its use is also associated with cardiovascular disease, respiratory changes, emphysema, and cancer [7]. It is commonly accepted that there are a multitude of variables that influence substance use, with complex interactions. These may be genetic, related to personality, or likened to differences in social circumstances and social responses [8].

It is commonly accepted that there are several individual factors that influence substance use. The literature often reports that gender and age are relevant explanatory factors of cannabis consumption. Thus, being male is usually outlined as an enabler of drug consumption [9,10,11,12,13,14,15,16,17,18,19,20,21,22]. Likewise, the probability of using cannabis increases with the adolescent’s age [9,12,18,21,22,23,24].

An adolescent’s family environment and the parental style often embed several factors that are linked with substance consumption [25]. Greater parental monitoring and support are related to a smaller probability of cannabis smoking [8,9,13,14,15,23,24,25,26,27,28,29]. Parental tolerance to substance use, or the parents’ habitual consumption, positively impact on adolescent drug use [2,9,13,17,18,22,23,26,27].

Any adolescent spends most of his/her time in school and with peers. Consequently, the literature reports that school engagement, academic success, and the influence of friends are relevant factors to explaining cannabis use. In this regard, whereas the authors of [2,8,13,20,30] outline academic performance and engagement in school as significant factors to explain drug consumption, the authors of [2,8,17,18,22,31,32,33,34,35,36] found that decisive behavior and the attitude of peers toward substance use explained cannabis consumption by teenagers.

There is a great deal of reports that indicate that belonging to a religious community diminishes both the chances of acquiring substances, and the opportunities to learn how to use them [37]. Nasim et al. [38] report that religion has a dissuasive effect on the risk behaviors of adolescents, which interferes with their use of drugs by helping them to internalize messages that discourage consumption. The significant protective effect of religiosity in cannabis smoking has been reported in samples from diverse cultures [8,9,11,12,14,15,19,20,24,26,30,31,35,37,38,39,40].

The literature on cannabis use by adolescents is wide. Thus, simple searches in the Scopus database show that this issue is currently a relevant topic in public health research. On 2 February 2022, the search, “cannabis AND adolescents AND factor AND use”, showed 26 scientific documents in 2022, 388 in 2021, and 440 in 2020. However, all of the reviewed literature performed quantitative analyses using conventional correlational methods, such as regression analysis. The novelty of this paper is the use of fuzzy set qualitative comparative analysis (fsQCA), which is presented by Ragin [40,41], to complement the results by ordered logistic regression (OLR). It must be outlined that the use of fsQCA in public health sciences is not new. Even though the authors of [40,42] suggest its use in health research, it is not common at all and, to the best of our knowledge, it has not been used in the assessment of the substance consumption of adolescents.

This paper evaluates the factors that induce the acceptance and rejection of cannabis use by analyzing a survey from Tarragona (Spain), with more than 1900 answers, that was conducted during 2019. In our study, the fsQCA and OLR are complementary rather than competitive since they focus the data analysis from two non-excluding points of view. The OLR is a variable-oriented technique, and so the odds ratios that is fitted with the OLR will allow us to measure the net incidence of each input variable on the use. On the other hand, fsQCA is case-oriented. Thus, fsQCA does not quantify with a coefficient the influence of the explanatory factors over the explained variable, but it will allow us to discover the profiles of adolescents that are consistently linked with cannabis consumption, as well as the combinations of factors that present significant linkages with cannabis rejection.

## 2. Materials and Methods

### 2.1. Sample and Survey

This cross-sectional study is based on the Planet Youth survey [43], to which secondary school students in Tarragona (Spain) submitted their responses. The adolescents who completed the questionnaire were 15 years (54%), 16 years (28.7%), and 17 years (16.8%) of age, and 0.5% of the responses were missed/refused. A total of 45.1% of the sample responded that they were male, 52.4% responded that they were female, and 3.5% refused to answer (responses were missed). The total number of adolescents that were interviewed was 1935 (from a population universe of N = 2407). The data were collected between February and March 2019.

With the help of social workers of the Tarragona city council, we asked school head teachers for their permission and help with the survey. The questionnaire, which included the questions in Table 1, was completed online in roughly 15–20 min. After receiving permission from the adolescents and their guardians, the teachers from the school checked that the adolescents understood the questions. Anonymity was fully ensured, as it was impossible for us to know which adolescent provided which response, as well as his/her identity. Informed consent was requested in an email that was sent to the adolescents’ parents or legal guardians, which asked that anyone who did not wish to allow their child to take part should contact the school office to have them excluded from the study. The research was approved by the authors’ institution (CEIPSA-2021-PDR-39).

Table 2 provides a detailed description of the relative frequencies of each possible response in every question. This table also shows the percentages of times the adolescents failed to provide an answer or preferred not to. After adjusting the sample because of the existence of failed answers/refused questions, we used 1750 observations.

The input variable (cannabis use (*USE*)) had seven possible answers. These answers were codified as: 1 = “never”; 2 = “1–2 times”; 3 = “3–5 times”; 4 = “6–9 times”; 5 = “10–19 times”; 6 = “20–39 times”; and 7 = “40 times or more”. That gradation was used, among others, by the authors of [6]. It is explained by using seven explanatory variables that were motivated in the introduction. Two of them are individual factors: gender (female and male) (GENDER) and age (AGE), which comprises 15, 16 and 17 years. We also consider two variables that are linked to the family environment: parental monitoring (MONITOR) and family tolerance to substance use (P_TOLER). Likewise, this paper also evaluates the impact of three variables that are linked to the adolescent’s social circumstances: his/her disengagement in school (DSCHOOL); his/her engagement in religion (RELIGION); and the substance use and deviant behavior of friends (PEER_USE). Table 1 and Table 2 show the number of items that each variable contains, and the Likert scale in which they were codified. In all the constructs, the items and gradations are the same as those used in [43]. Table 3 indicates the hypothetical impact of the input variables on cannabis consumption, based on the literature that is reviewed in the introduction. It will be applied to conduct the fsQCA, as well as to develop the discussion section.

### 2.2. Analytical Procedure

The use of the OLR and fsQCA in our study is complementary since they allow the analysis of data from two non-excluding points of view. OLR fits an analytical equation that quantifies the effect of the input factors on the explained variable. On the other hand, fsQCA displays several combinations (recipes) of the input variables that may produce a given output by means of a Boolean function.

Whereas regression analysis is a variable-oriented technique, fsQCA is case-oriented. It measures the membership degree of each case in the set of attributes and the outcome set by using fuzzy set union and intersection operators [44]. Thus, fsQCA allows for the discovery of the several ways in which the input variables combine to produce an output [45].

In regression analysis, an input variable can only be linked with an output with one sign. On the other hand, fsQCA allows different signs for the influence of an input factor on the output variable in two combinations of explanatory variables. This property could be useful for our purposes. For example, it is commonly accepted that religious activities usually have a protective capability with regard to cannabis use. Thus, a configurational analysis may show that, in some prime implicates, the existence (non-existence) of religiosity leads to the non-use (use) of cannabis. However, certain kinds of spirituality could be an enabler of marijuana smoking [46,47]. This fact might induce configurations where the existence of a religious feeling is also a condition of cannabis use.

Likewise—and contrary to correlational methods—fsQCA does not assume symmetrical relationships between the variables, despite being effective in this case [48]. This is relevant because the combinations of factors that produce acceptance and rejection are not necessarily non-symmetrical.

Data handling will embed a statistical analysis, which includes OLR, and fsQCA that, following [48], uses some of the results that are obtained in the statistical analysis.

#### 2.2.1. Statistical Analysis

Previous to the application of fsQCA, we perform a conventional statistical analysis of the impact of the assessed factors on cannabis consumption, as follows:

Step 1: We measure the reliability of the variable (DSCHOOL, MONITOR, RELIGION, P_TOLER, PEER_USE) scales by using Cronbach’s alpha. All items and scales are taken from [43]. The scale validation in our sample is an intermediate step in the implementation of not only OLR, but also fsQCA [48].

Step 2: We define the input variables in terms of the membership functions in such a way that, for the ith variable ($X_{i}$), the value of the *j*th observation ($x_{i,j}$) is transformed to $m_{X_{i,j}}$, as follows:

GENDER = Dichotomous variable that takes 0 for males and 1 for females.

AGE = Variable that takes 0, 0.5, and 1 if the respondent is 15 years, 16 years, and 17 years old, respectively.

As far as multiple-item variables (such as DSCHOOL, MONITOR, RELIGION, P_TOLER and PEER_USE) are concerned; we summed the evaluations of each item. It is a simple but usual way to deal with this question in empirical research [49]. Notice that, in these sums, DSCHOOL ranks from 10 to 50; MONITOR from 11 to 44; RELIGION from 12 to 48; P_TOLER from 3 to 12; and PEER_USE from 5 to 25.

Subsequently, we obtained the membership evaluations of these variables by using a common procedure in fsQCA [41].

(1) $$m_{x_{i,j}}=\left\{\begin{array}{c} \begin{array}{c} \begin{array}{cc} 0\; & x_{i,j}\leq X_{i}^{10th} \end{array}\\ \begin{array}{cc} \frac{x_{i,j}-X_{i}^{10th}}{2\left(X_{i}^{50th}-X_{i}^{10th}\right)} & X_{i}^{10th}<x_{i,j}\leq X_{i}^{50th} \end{array} \end{array}\\ \begin{array}{cc} 0.5+\frac{x_{i,j}-X_{i}^{50th}}{2\left(X_{i}^{90th}-X_{i}^{50th}\right)} & X_{i}^{50th}<x_{i,j}\leq X_{i}^{90th} \end{array}\\ \begin{array}{cc} \;1\; & x_{i,j}>X_{i}^{90th} \end{array} \end{array}\right.$$

where $X_{i}^{sth}$ the *s*th quantile in the sample of the *i*th variable. Following Ragin [41], we have chosen for the thresholds the 10th, 50th, and 90th percentiles.

Step 3. We run the contrarian case analysis that is described in [48], which will allow us to state a preliminary sign and the statistical significance of the impact of the input variables on the *USE*. In this step, the values of the input variables are those that are defined in Step 2. The input variable (*USE*) is quantified by simply taking its value on the Likert scale (in Table 1 and Table 2) for its *j*th observation, *y_j_*, which can take values within {1,2,…,7} (see Table 2). Subsequently, we divide the sample by using the quintiles of the variables, and we then build up cross-tabulations of the *USE* with respect to the input variables across these quintiles. Crosstabs allow for stating not only the main effect between every input on the output, and its statistical significance, but also the existence of cases outside of the main effect that justify a configurational analysis. In this regard, Pappas and Woodside [48] advice using the Phi indicator to measure the weight of observations out of principal relation. The Phi measures the significance of the statistical relation of the variables that are embedded in the crosstab analysis, but not its direction. A Phi^2^ < 0.5 suggests the existence a number of cases that are out of the mainstream relations between the input factors, and the explained variable is enough to justify a configurational analysis.

Step 4. We fit the output with an ordered linear regression (OLR) model with the help of Gretl software [50]. Meanwhile, the *USE* is defined on a Likert scale that ranges from 1 to 7 (see Table 1 and Table 2), and the input variables are quantified following Step 2. At this step, we only consider the direct effect of each variable on the *USE*. The goodness of fit with the data is measured with McFaddens’ pseudo R^2^, and the significance of the overall model is stated with the log-likelihood ratio (LR). The significance of the odd ratios will be tested throughout the z-scores of their natural logarithms.

#### 2.2.2. Fuzzy Set Qualitative Comparative Analysis

We perform fsQCA by following the following steps:

Step 1. The membership functions of the input variables are those that are defined in Step 2. For the output variable (*USE*), from the *j*th observation (*y_j_*∈{1,2,3,…,7}), we define:

$$m_{USE_{j}}=\left\{\begin{array}{c} \begin{array}{cc} 1 & \;y_{j}\geq 5 \end{array}\\ \begin{array}{cc} 0.9 & y_{j}=4 \end{array}\\ \begin{array}{cc} 0.8 & y_{j}=3 \end{array}\\ \begin{array}{cc} 0.2 & y_{j}=2 \end{array}\\ \begin{array}{cc} 0 & y_{j}=1 \end{array} \end{array}\right.$$

Step 2. We implement the fsQCA with fsQCA 3.1 software [51]. This enables finding logical implications that fit the output results by running a Boolean minimization algorithm. If we symbolize the negation of a variable as, “~”, we independently adjust the Boolean functions:

*USE* = f(GENDER, AGE, DSCHOOL, MONITOR, RELIGION, P_TOLER, PEER_USE) (2)

~*USE* = f(GENDER, AGE, DSCHOOL, MONITOR, RELIGION, P_TOLER, PEER_USE) (3)

Therefore, (2) explains cannabis use, and (3) explains cannabis rejection. With regard to this, it must be outlined that the membership degree in the negated variable (~*X_i__,_*) is $m_{{-X}_{ij}}=1-m_{X_{ij}}$. Thus, for ~*USE*, we state: $m_{-USE_{j}}=1-m_{USE_{j}}$.

The adjustment of *USE* and ~*USE* is performed with the Quine–McCluskey algorithm to find the essential prime implicates (configurations or recipes) in the truth table. These implicates conform to the so-called qualitative comparative analysis complex solution (CS).

Step 3. The CS is usually hard to interpret since it is built up with no more assumptions than the data. Therefore, fsQCA 3.1 also offers a parsimonious solution (PS). It is fitted by applying the Quine–McCluskey algorithm and any remainder over a non-observed configuration of the variables in order to make the solution as easy as possible [41].

Step 4. To continue the minimization process, it must be supposed for non-observed configurations if an input variable contributes to the output exclusively when it is present, absent, or in both cases, by using well-founded hypotheses. This step allows us to obtain the so-called intermediate solution (IS) [41]. In our paper, we use the hypotheses in Table 3 that are supported by the literature revised in the introduction.

For an in-depth explanation of the Boolean minimization procedures in the CS, PS, and IS, see [52].

Step 5. To measure the explanatory power of a given recipe, its consistency (*cons*) and coverage (*cov*) must be calculated. Let it be a possible prime implicate (configuration or recipe) (*Z*) that, without a loss of generality, we built as:

$$Z=X_{1}*X_{2}*\ldots *X_{r}$$

where 1 ≤ *r* ≤ *n*, where *n* is the number of output variables, and “*” stands for the Boolean product. Thus, we can obtain for the *j*th observation:

$$m_{Z_{j}}=\min\{m_{X_{1,j}};\;m_{X_{2,j}};\;\ldots ;\;m_{X_{r,j}}\}$$

Therefore, the consistency of the recipe (*Z*) in producing an output (*Y*) is:

(4) $$Cons_{Z\to Y}=\frac{\sum_{j}\min\left\{m_{Z_{j}};m_{Y_{j}}\right\}}{\sum_{j}m_{Z_{j}}}$$

Subsequently, the coverage of the recipe (*Z*) to produce *Y* is:

(5) $$Cov_{Z\to Y}=\frac{\sum_{j}\min\left\{m_{Z_{j}};m_{Y_{j}}\right\}}{\sum_{j}m_{Y_{j}}}$$

The consistency measures the membership degree of a combination of causes (a recipe) within the outcome set. It is similar to a statistical measure of significance [53]. There is a wide consensus that, in order to consider an essential prime that is implicated as a sufficient condition, *cons* > 0.75 (or better, *cons* > 0.8). The coverage measures the proportion of outcomes that are explained by a recipe (i.e., it is a measure of the empirical relevance, similar to the R^2^ [53].

Step 6. To assess how the input variables and their combinations impact on the acceptance and rejection of cannabis use, the solutions from the fsQCA must be interpreted. There is no unified point of view about what solution (CS, PS, or IS) to take into account. The CS uses only empirical data, but the recipes in that solution are often hard to interpret. In this regard, the authors of [41] propose combining both the IS and PS to the state core (from the PS) and peripheral (present only in the IS) conditions.

## 3. Results

### 3.1. Results of Statistical Analysis

When validating the scales (Table 4), we checked that all of the constructs presented a Cronbach’s alpha > 0.7. Thus, we have robust evidence on the internal consistency of DSCHOOL, MONITOR, RELIGION, P_TOLER, and PEER_USE. Thus, in the subsequent analysis, these variables are defined following (1). The 10th, 50th, and 90th quantiles that are needed to fit (1) are displayed in Table 4.

Table 4 also shows that the values that are attained by the Phi measure show a significant relation of the *USE* to all the explanatory variables (*p* < 0.0001 in all cases, except for RELIGION, where *p* = 0.0001). Kendall’s Tau-b correlation indicates a positive (negative) linkage of AGE, P_TOLER, and PEER_USE (GENDER, DSCHOOL, MONITOR, RELIGION) with *USE*, where *p* < 0.0001 in all the cases.

The results of the OLR, in Table 5, allow for nuancing the results in Table 4. We found that the ordered logit model was significant (LR = 2002; *p* < 0.0001). Likewise, the pseudo-R^2^ = 48.27%. Even though the determination of a given R^2^ as good or less good is quite subjective, in an ordered logistic regression context, a pseudo R^2^ greater than 25% has been qualified by several authors, at least, as good [54]. We observed a significant negative average influence of: being female (odd ratio (OR) = 0.383; *p* < 0.0001; 95% CI = [0.29, 0.51]); MONITOR (OR = 0.587; *p* = 0.0201; 95% CI = [0.37, 0.92]); and RELIGION (OR = 0.476; *p* = 0.0006; 95% CI = [0.31, 0.73]). Thus, those factors must be considered as inhibitors of cannabis consumption. We have identified, as risk factors, family permissiveness (OR = 42.01; *p* < 0.0001; 95% CI = [28.45, 62.04]) and PEER_USE (OR = 5.60; *p* < 0.0001; 95% CI = [3.26, 9.63]). The sign of the relation of AGE (OR = 1.168; *p* = 0.4809; 95% CI = [0.76, 1.80]) and DSCHOOL (OR = 0.755; *p* = 0.2402; 95% CI = [0.47, 1.21]) fitted with the Tau-b correlation but did not show statistical significance.

### 3.2. Results of fsQCA

Table 4 shows the results of the Phi measure from a contrarian case study. With the exception of parental tolerance, all the linkages between the input factors and substance use attain Phi^2^ < 0.5. This fact could indicate that there is a significant number of cases that fail out of the mainstream relation, and therefore the convenience of a configuration analysis by means of fsQCA is justified [47].

Table 6 shows the results of the fsQCA for the use and non-use of cannabis. With regard to this, we must outline that the CS, IS, and PS for use and non-use are the same. It can be observed that non-use is clearly better explained than use. The consistency is 0.952 for non-use vs. 0.808 for use, and the coverage attains 0.869 for non-use and 0.357 for use.

As far as *USE* is concerned, we can observe that:

1. The configurational analysis detects that the confluence of being male and perceiving a parental tolerance toward the use of substances is present in all the explanatory configurations. It is also very relevant in the presence of PEER_USE (3 over 4 recipes);
2. The absence of religiousness and being older is a condition in two prime implicates of *USE*;
3. Parental monitoring is present with a contradictory sign in two recipes. The third recipe shows a group of adolescents whose cannabis use is explained by the confluence of high parental monitoring and other variables. However, there also exists a part of the responses that acknowledges cannabis smoking that can be explained by the confluence of low parental monitoring with other factors (fourth recipe);
4. The absence of DSCHOOL, then, which can be understood as well-being in school, is present in one configuration that explains use.

With regard to the configurations fitted for non-users, we observe the following patterns:

5. The perception of great disagreement toward substance use by family is a sufficient condition to be a nonconsumer (*cons* = 0.956, *cov* = 0.861);
6. The McCluskey algorithm identifies two more prime implicates with great *cons* (≈0.95) but limited coverage (<0.15) that are not linked with the attitude of the family toward the substance. In these prime implicates, being female, having a high degree of parental monitoring, religiousness, and the absence of PEER_USE must be present with non-disengagement in school (in the second recipe) and greater ages (in the third).

## 4. Discussion

In this paper, we used ordered logistic regression (OLR) and fuzzy set qualitative comparative analysis (fsQCA) to assess the factors that may explain the adolescent use of cannabis (*USE*). OLR allows for the identification of the relevant variables at the population level, and the interpretation of the strengths of their risk/protection capabilities, according to the value of their odd ratios.

The OLR findings reveal that being female (GENDER), a high parental implication in adolescent activities (MONITOR), and religiousness (RELIGION) have significant protective impacts against cannabis smoking habits. The parents’ or legal guardians’ liberal attitude toward substance consumption (P_TOLER), and the habitual use of substances and the conduct deviance of peers (PEER_USE) are significant enablers of cannabis smoking. These facts are in accordance with what we expected, and they are displayed in Table 3. The OLR does not detect a significant influence of the age (AGE) and the adolescents’ disengagement in school (DSCHOOL) on *USE*. These findings contradict the supposed influence of these factors on use that is outlined in Table 3.

The fact that being male is a significant risk factor of cannabis use is in accordance with the mainstream findings of the literature [6,9,10,11,12,13,14,15,16,17,18,19,20,21,22,29]. The significant and negative relation of MONITOR with *USE* is widely supported by the reviewed papers, which found similar results for the influence of the parental monitoring on the drug consumption. Analogous conclusions are extracted from OLR findings on the impact of RELIGION on *USE*, and from the reviewed literature [8,9,11,12,14,15,19,20,21,22,30,31,35,37,38].

As we expected, our results display positive and significant influences of P_TOLER and PEER_USE on *USE*, whereas, in the case of P_TOLER, our results confirm those in [6,9,13,17,18,22,23,26,27], and our findings on PEER_USE are in line with those of [8,17,18,22,25,31,32,33,34,35,36].

Using fsQCA on the data allows for the extraction of complementary conclusions from the sample to those by the OLR. It discovered consistent patterns of at-risk and out-of-risk substance use among adolescents. Moreover, it also allowed for an evaluation of the symmetry degree of the impact on the cannabis acceptance and cannabis rejection by the assessed factors.

With regard to the risk profiles, the fsQCA identified four prime implicates. In all these profiles, being male and parental tolerance are present. Likewise, PEER_USE is a condition in three recipes. The rest of the variables play secondary roles. Likewise, the fsQCA also identified three profiles that are linked with a consistent resistance to cannabis consumption. The first profile consists simply in parental intolerance to substance use. The other two prime implicates always combine being female, a great degree of parental monitoring, religious engagement, and the absence of consumption by peers.

The implementation of the fsQCA also found clear asymmetries present in the explanations of acceptance and rejection to cannabis consumption. With regard to this, we can outline that:

(a) The explanatory factors have an asymmetric adjustment capability over the use and non-use. Whereas resistance to cannabis is extremely well explained (*cons* = 0.95 and *cov* = 0.86), cannabis use attains an acceptable consistency (*cons* = 0.81) and not-so-great coverage (*cov* = 0.36). Thus, the satisfactory OLR determination coefficient is because the factors are significantly capable of explaining cannabis non-use, but are not as capable at explaining use;
(b) The parental attitude toward substance use is the key factor in adolescent cannabis smoking habits. However, the impact of P_TOLER is asymmetrical on use and non-use. To attain a significant consistency to produce use, P_TOLER always needs to be combined with being male and, depending on the configuration, with variables such as, e.g., PEER_USE and ~RELIGION. On the other hand, the absence of P_TOLER is a sufficient condition to reject cannabis consumption;
(c) MONITOR is relevant to explain cannabis use, but much less relevant than parental tolerance. This finding is consistent with [23]. Likewise, whereas the sign of MONITOR is not unique when it takes part in the explanatory recipes of *USE* (it appears in two different recipes: affirmed and negated), it shows a consistent positive relation with non-use (it is present in two recipes explaining non-use). Therefore, the sign of MONITOR in OLR can be produced basically by its impact on cannabis non-use;
(d) The presence of AGE is necessary in two recipes that explain *USE*. That is to say, the fsQCA positively linked older ages with cannabis consumption. This is in accordance with [9,12,18,21,22,23,24]. However, notice that this impact is asymmetrical on non-use since AGE is also present in a recipe that explains that behavior. That is to say, AGE does not have a univocal sign in its relation to consumption;
(e) GENDER, RELIGION, and PEER_USE exhibit consistent signs in their relations with cannabis smoking. GENDER and RELIGION (PEER_USE) are always negated (affirmed) in the explanatory prime implicates of *USE*, and affirmed (negated) when it induces cannabis rejection. Likewise, GENDER and PEER_USE participate in more explanatory recipes of use and non-use than RELIGION. Therefore, we can consider the impact of these variables on cannabis consumption near to symmetrical.

## 5. Conclusions

### 5.1. Practical Implications

To the best of our knowledge, a combinatorial analysis of the variables that induce the acceptance of and the unwillingness to partake in substance use by adolescents has not been performed. This paper shows that the fsQCA not only helped to understand how the input variables combine to influence the consumption and inhibition of cannabis smoking, which is a relevant question in this substance use [29], but also that some of the assessed factors impact asymmetrically on the substance use and non-use. These findings have fair potential implications for health policies. On the one hand, we have identified several adolescent profiles that are consistently linked with cannabis smoking and that are, thus, of great concern. On the other hand, we have also determined adolescent profiles that are very compatible with the consistent rejection of cannabis. Thus, these kinds of adolescents need lower amounts of care with regard to cannabis consumption. Likewise, it seems that the parental attitude toward substances is the keystone that explains use and non-use. This fact suggests that cannabis prevention campaigns by health policy makers must be addressed not only to adolescents, but also to their families.

### 5.2. Limitations and Further Research

This study is based on a cross-sectional survey. Although our conclusions about the impact of the assessed factors generally reinforce the findings in other countries, further research will require longitudinal data to assess the directionality of the influences. Tarragona is a city of nearly 150,000 citizens, whose main economic activities are industry and services, and with a relevant portion of the migrant population from Magreb and Latin America. Likewise, our sample is very comprehensive of the universe of study (1750 useful responses in a universe of 2407 teenagers). Therefore, we feel that the results could be representative for similar social environments in Spain, such as cities within the area of influence of Barcelona or Madrid. On the other hand, we believe that the results might be hard to transfer to adolescents from rural Spanish regions, whose main activities are the primary sector or tourism, and which have low proportions of immigrant citizens, and which are clearly socioeconomically different. Of course, these facts also depend on other different formal factors that are inherent to the sample and the experiment designs, such as, for example, the prevalence in the use of cannabis.

An additional drawback is the not-so-great coverage of the fsQCA for cannabis use. Although the recipes that are linked to substance consumption have an adequate consistency, the coverage of each recipe, and that by the overall intermediate solution (not greater that 0.4), reveals that the risk profiles are not extremely comprehensive.

In our opinion, the use of conjointly correlational methods, such as regression and fsQCA, will also allow for the obtainment of useful empirical results in samples from other countries and cultures, in adult populations, or in different substance use phenomena (e.g., alcohol abuse, other drugs, or multiple-substance consumption). It will allow for the discovery of not only the significant risk/protective factors, but also of the risk profiles that merit high concern, as well as of low-risk profiles. We feel that these questions could be very useful for guiding public health policies.

## Figures and Tables

**Table 1:** Items of the questionnaire.

OUTPUT VARIABLE	
Cannabis use (*USE*): How often (if ever) have you used Cannabis (hashish or marijuana) last 30 days?	
**INPUT VARIABLES**	
GENDER: Are you a boy or a girl?	
AGE: What is your age?	
Disengagement to school (DSCHOOL). How well do the following statements apply to you? (From 1 = almost never to 5 = almost always)	
DSCHOOL1 =	I find the school studies pointless
DSCHOOL2 =	I am bored with the studies
DSCHOOL3 =	I am poorly prepared for classes
DSCHOOL4 =	I feel I do not put enough effort into the studies
DSCHOOL5 =	I find the studies too easy
DSCHOOL6 =	I find the studies too difficult
DSCHOOL7 =	I feel bad at school
DSCHOOL8 =	I want to quit school
DSCHOOL9 =	I want to change the school
DSCHOOL10 =	I get on badly with the teachers
Parental monitoring (MONITOR). How well do the following statements apply to you? (From 1 = very poorly to 4 = very well)	
MONITOR1 =	My parents find it important that I do well in my studies
MONITOR2 =	My parents set definite rules about what I can do at home
MONITOR3 =	My parents set definite rules about what I can do outside
MONITOR4 =	My parents set definite rules about when I should be home in the evening
MONITOR5 =	My parents know whom I am with in the evenings
MONITOR6 =	My parents know where I am in the evenings
MONITOR7 =	My parents know my friends
MONITOR8 =	My parents know the parents of my friends
MONITOR9 =	My parents often talk to the parents of my friends
MONITOR10 =	My parents and the parents of my friends sometimes meet to talk to one another
MONITOR11 =	My parents follow what I do in my recreational time
Religiousness (RELIGION). How well do the following statements apply to you? (From 1 = very poorly to 4 = very well)	
RELIGION1 =	I believe in God
RELIGION2 =	My faith is important to me
RELIGION3 =	I pray to god on a regular basis
RELIGION4 =	I regularly read in the scriptures of my faith
RELIGION5 =	I regularly attend religious services
RELIGION6 =	I regularly take part in religious activities other
RELIGION7 =	I would be able to get support from god if I needed
RELIGION8 =	I have sought support from god when I have needed it
RELIGION9 =	My best friends are religious
RELIGION10 =	Most of my acquaintances are religious
RELIGION11 =	My mother (foster/stepmother) is religious
RELIGION12 =	My father (foster/stepfather) is religious
Tolerance padres/legal guardians (P_TOLER). How do you think your parents would react if you did any of the following? (From 1 = Totally against to 4 = not care)	
P_TOLER1 =	If you would smoke cigarettes
P_TOLER2 =	If you would become drunk
P_TOLER3 =	If you would smoke cannabis
Substance use by peers (PEER_USE): How many peers/friends do you think do the following? (From 1 = none to 5 = almost all)	
PEER_USE1 =	Smoke cigarettes
PEER_USE2 =	Drink alcohol (beer, wine, or spirits)
PEER_USE3 =	Become drunk at least once a month
PEER_USE4 =	Smoke hash or marijuana
PEER_USE5 =	Pick fights or search out for fights

**Table 2:** Response relative frequencies for every question in the survey (in percentages).

*USE* (OUTPUT VARIABLE)						
1 = ”Never”	2 = “1–2”	3 = “3–5”	4 = “6–9”	5 = “10–19”	6 = “20–39”	7= “40 times or +”
72.52	8.14	4.51	3.08	3.30	2.31	6.27
**INPUT VARIABLES**						
Applies:	1 = almost never		2 = seldom	3 = sometimes	4 = often	5 = almost always
DSCHOOL1	31.64		27.51	25.54	10.55	4.86
DSCHOOL2	12.93		25.03	31.44	19.23	11.48
DSCHOOL3	36.14		29.91	18.90	9.24	5.82
DSCHOOL4	15.85		21.76	28.08	21.04	13.16
DSCHOOL5	29.91		32.92	24.51	8.83	3.95
DSCHOOL6	15.22		27.02	33.23	17.81	6.73
DSCHOOL7	60.29		16.84	11.12	6.76	4.99
DSCHOOL8	71.01		11.16	8.34	4.59	5.01
DSCHOOL9	66.11		11.95	9.25	5.51	7.28
DSCHOOL10	50.00		27.12	13.35	5.49	4.04
Applies:	1 = very poorly			2 = poorly	3 = well	4 = very well
MONITOR1	0.83			2.17	19.36	77.74
MONITOR2	3.73			16.08	46.58	33.71
MONITOR3	6.28			19.98	43.72	29.92
MONITOR4	6.35			17.27	37.25	39.02
MONITOR5	3.55			8.45	27.11	60.79
MONITOR6	2.71			7.72	24.43	65.03
MONITOR7	2.40			8.85	35.73	53.02
MONITOR8	8.86			26.28	40.46	24.40
MONITOR9	20.31			31.25	33.54	14.90
MONITOR10	36.29			32.53	20.75	10.43
MONITOR11	19.90			29.06	33.44	17.60
Applies:	1 = very poorly			2 = poorly	3 = well	4 = very well
RELIGION1	54.20			16.47	13.09	16.25
RELIGION2	47.70			18.42	14.58	19.30
RELIGION3	69.79			14.44	7.28	8.60
RELIGION4	79.25			9.55	6.04	5.16
RELIGION5	76.75			11.07	5.70	6.47
RELIGION6	77.23			11.77	5.17	5.83
RELIGION7	63.77			13.77	11.67	10.68
RELIGION8	57.87			15.84	13.31	12.98
RELIGION9	54.25			27.40	12.27	6.08
RELIGION10	45.25			27.92	19.09	7.84
RELIGION11	48.06			15.50	15.39	21.15
RELIGION12	55.11			14.33	14.11	16.44
	1 = totally against			2 = much against	3 = rather against	4 = not care
P_TOLER1	65.59			17.48	11.47	5.35
P_TOLER2	47.53			20.52	23.09	8.86
P_TOLER3	82.94			10.89	4.04	2.13
	1 = none		2 = a few	3 = some	4 = most	5 = almost all
PEER_USE1	25.93		25.37	27.73	14.32	6.65
PEER_USE2	17.70		16.80	23.56	25.37	16.57
PEER_USE3	28.44		23.14	24.60	15.24	8.47
PEER_USE4	41.08		25.96	18.51	8.92	5.53
PEER_USE5	67.65		19.68	8.82	2.15	1.70

Note: In the output variables, the codifications from 1 to 7 quantify the number of times of cannabis use in the last 30 days.

**Table 3:** Hypotheses used to find intermediate solutions.

Variable	Hypothesis on the Influence on Cannabis Use
GENDER	Females tend to consume less cannabis than males.
AGE	Older ages are more exposed to cannabis consumption.
DSCHOOL	Disengagement in school is linked with cannabis use.
MONITOR	Greater parental monitoring is linked with non-use.
RELIGION	Religiousness has been found to be a protective factor against use.
P_TOLER	Family tolerance to substance use is linked with cannabis consumption.
PEER_USE	Peers’ use and conduct deviance is linked with substance use.

**Table 4:** Cronbach’s alphas of scales, measures of association between independent variables and use, and 10, 50, and 90% quantiles of the variables: DSCHOOL, MONITOR, RELIGION, P_TOLER, and PEER_USE.

Variable	Cronbach-α	Phi	*p*-Value	Tau-b	*p*-Value	Rank	10% Quantile	50% Quantile	90% Quantile
GENDER		0.125	<0.0001	−0.101	<0.0001				
AGE		0.173	<0.0001	0.165	<0.0001				
DSCHOOL	0.769	0.166	<0.0001	−0.127	<0.0001	[10, 50]	28	39	45
MONITOR	0.799	0.243	<0.0001	−0.197	<0.0001	[11, 44]	15	22	29
RELIGION	0.928	0.121	0.001	−0.089	<0.0001	[12, 48]	12	17	34
P_TOLER	0.753	0.931	<0.0001	0.749	<0.0001	[3, 12]	3	3	9
PEER_USE	0.884	0.5	<0.0001	0.396	<0.0001	[5, 25]	5	11	18

**Table 5:** Ordered logistic regression estimates.

	OR	OR Logarithm	Std. Dev.	*z*-Ratio	*p*-Value	95% CI (OR)
GENDER	0.383	−0.960	0.148	−6.471	<0.0001	[0.29, 0.51]
AGE	1.168	0.155	0.220	0.7049	0.4809	[0.76, 1.80]
DSCHOOL	0.755	−0.281	0.239	−1.175	0.2402	[0.47, 1.21]
MONITOR	0.587	−0.533	0.229	−2.325	0.0201	[0.37, 0.92]
RELIGION	0.476	−0.742	0.217	−3.415	0.0006	[0.31, 0.73]
P_TOLER	42.010	3.738	0.689	18.79	<0.0001	[28.45, 62.04]
PEER_USE	5.600	1.723	0.276	6.232	<0.0001	[3.26, 9.63]
LR ratio = 2002.91 (*p* < 0.0001)						
Pseudo R^2^ = 48.26%						

**Table 6:** fsQCA solutions of *USE* and ~*USE*.

	*USE*				NON-*USE* (~*USE*)			
Factor|Recipe	1	2	3	4	Factor|Recipe	1	2	3
GENDER	⊗	⊗	⊗	⊗	GENDER		•	•
AGE		•		•	AGE			•
DSCHOOL			⊗		DSCHOOL		⊗	
MONITOR			•	⊗	MONITOR		•	•
RELIGION	⊗			⊗	RELIGION		•	•
P_TOLER	•	•	•	•	P_TOLER	⊗		
PEER_USE	•	•	•		PEER_USE		⊗	⊗
cons	0.831	0.819	0.774	0.787	cons	0.956	0.949	0.941
cov	0.260	0.300	0.173	0.127	cov	0.861	0.135	0.137
*cons* of IS	0.808				*cons* of IS	0.952		
*cov* of IS	0.357				*cov* of IS	0.869		

Note: A full circle (•) indicates the presence of a condition, and circles with x (⊗) indicate its absence.

## Data Availability

Data available upon request.
